# Aerolysin gene characterization and antimicrobial resistance profile of *Aeromonas hydrophila* isolated from milkfish (*Chanos chanos*) in Gresik, Indonesia

**DOI:** 10.14202/vetworld.2022.1759-1764

**Published:** 2022-07-23

**Authors:** Faisal Fikri, Dhandy Koesoemo Wardhana, Agus Purnomo, Shafia Khairani, Shekhar Chhetri, Muhammad Thohawi Elziyad Purnama

**Affiliations:** 1 Department of Veterinary Science, Division of Veterinary Clinical Pathology and Physiology, Faculty of Veterinary Medicine, Universitas Airlangga, Surabaya, Indonesia; 2 Department of Veterinary Science, School of Health and Life Sciences, Universitas Airlangga, Surabaya, Indonesia; 3 Department of Veterinary Science, Division of Veterinary Public Health, Faculty of Veterinary Medicine, Universitas Airlangga, Surabaya, Indonesia; 4 Department of Veterinary Surgery and Radiology, Faculty of Veterinary Medicine, Universitas Gadjah Mada, Yogyakarta, Indonesia; 5 Department of Biomedical Science, Faculty of Medicine, Universitas Padjajaran, Bandung, Indonesia; 6 Department of Animal Science, College of Natural Resources, Royal University of Bhutan, Lobesa, Punakha, Bhutan; 7 Department of Veterinary Science, Division of Veterinary Anatomy, Faculty of Veterinary Medicine, Universitas Airlangga, Surabaya, Indonesia

**Keywords:** aerolysin gene, *Aeromonas hydrophila*, antimicrobial resistance, milkfish, public health

## Abstract

**Background and Aim:**

Motile Aeromonas septicemia is a crucial disease in freshwater fish. Aeromonas hydrophila is a disease agent associated with sporadic fish mortality, food safety, and public health. This study aimed to estimate the prevalence and the presence of the aerolysin gene and antimicrobial resistance profile of A. hydrophila isolated from milkfish in Gresik, Indonesia.

**Materials and Methods:**

A total of 153 milkfish gill samples were collected from 16 locations in Gresik and then cultured and identified using biochemical tests. The aerolysin gene was investigated using a polymerase chain reaction, and antimicrobial resistance profiles of the recovered isolates were investigated.

**Results:**

Of the 153 examined samples, 35 (22.9%) were confirmed positive for A. hydrophila and 22 (62.9%) presented the aerolysin gene. The recovered isolates were resistant to the following antibiotics: Amoxicillin (62.9%), tetracycline (60%), streptomycin (54.3%), cefotaxime (51.4%), gentamycin (31.4%), kanamycin (28.6%), erythromycin (25.7%), chloramphenicol (20%), and trimethoprim (14.3%). Meanwhile, only ciprofloxacin, nalidixic acid, and imipenem were indicated as susceptible.

**Conclusion:**

The presence of the aerolysin gene is vital in determining the virulence of A. hydrophila. The study results indicated a high aerolysin gene prevalence. In addition, this study emphasized antibiotic use monitoring, food safety improvement, and negative impact reduction on human health and the environment.

## Introduction

Infectious and parasitic disease control in fish is a factor that determines the aquaculture business sustainability levels [1]. Milkfish (*Chanos chanos*) is a freshwater fish that has a high commodity level in Gresik, Indonesia. *Aeromonas hydrophila* is one of the Gram-negative bacteria that potentially and massively infect milkfish aquaculture. *A. hydrophila* infection can occur in high stocking densities, high temperatures, high organic matter, and even in well-maintained ponds. Extreme environments can trigger stress levels in fish and increase the risk of aquaculture-reared fish infections [2].

*A. hydrophila* optimally grows at a maximum temperature of 38–41°C and a minimum of 0–5°C at a pH of 5.5–9 and reproduces asexually or binary fission with cell elongation followed by nuclear division [3]. *A. hydrophila* has a habitat in estuarine and freshwater areas, and its presence is related to the content of organic matter or aquatic sediments. In addition, *A. hydrophila* is found in tropical and subtropical areas [4]. Its infection often occurs in the dry season because of the relatively high organic matter content in the waters. *A. hydrophila* plays a role in the decomposition of organic matter; thus, it is often observed in reared water [5].

Acute infection can be mediated through wounds, the digestive tract, and gills, then spreads in the blood vessels and causes hemorrhagic septicemia [6]. The study conducted by Rasmussen-Ivey *et al*. [7] reported that *A. hydrophila* has components of hemolysin, cytotoxic enterotoxin, lipase, and aerolysin (*aer-A*) genes that cause acute hemorrhagic septicemia. Inappropriate use of antibiotics has implications for the incidence of antimicrobial resistance, especially multidrug resistance, which is a current issue. Further, the use of antibiotics has the potential to increase *A. hydrophila* resistance in addition to polluting the environment and being expensive [8].

In general, bacteria can resist various antibiotics and enrich their virulence features. Antimicrobial *A. hydrophila* resistance is a global problem due to antibiotic misuse [9]. Thus, this study aimed to estimate the prevalence of *A. hydrophila* isolated from milkfish in Gresik, Indonesia. In addition, the characterization of the *aer-A* gene and antimicrobial resistance profile was emphasized.

## Materials and Methods

### Ethical approval

This study was approved by the Ethical Committee of Animal Care and Use, Universitas Airlangga, with reference No.388/HRECC.FODM/III/2020.

### Study period and location

**Figure-1:**
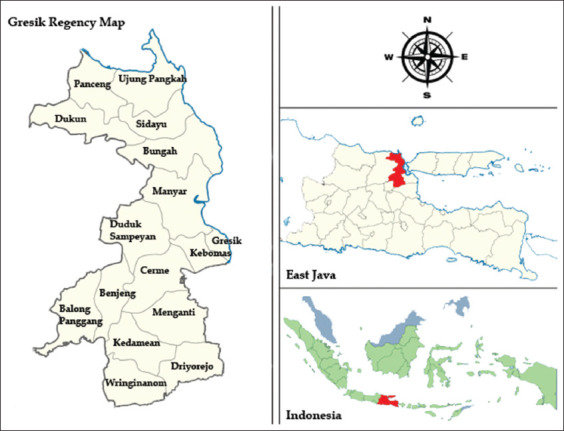
Gresik regency map [Source: https://doi.org/10.6084/m9.figshare.19103168.v2].

This study was performed for 5 months (March-July 2020). The milkfish samples were collected from Dukun (6°58’44.0”S 112°26’24.5”E) (n = 7), Panceng (6°56’29.8”S 112°27’56.7”E) (n = 8), Ujung Pangkah (6°52’49.9”S 112°33’11.7”E) (n = 11), Sidayu (6°58’18.4”S 112°35’17.6”E) (n = 15), Bungah (7°03’19.1”S 112°34’34.9”E) (n = 14), Manyar (7°07’21.2”S 112°36’14.5”E) (n = 11), Gresik (7°09’01.8”S 112°39’11.8”E) (n = 12), Kebomas (7°09’59.1”S 112°38’17.5”E) (n = 7), Duduk Sampeyan (7°07’38.2”S 112°31’49.4”E) (n = 8), Cerme (7°12’24.7”S 112°34’36.9”E) (n = 11), Benjeng (7°15’02.9”S 112°29’09.2”E) (n = 10), Balong Panggang (7°15’39.3”S 112°25’54.4”E) (n = 7), Wringinanom (7°21’24.6”S 112°31’12.1”E) (n = 8), Menganti (7°17’34.9”S 112°35’07.9”E) (n = 8), Kedamean (7°19’20.5”S 112°33’57.6”E) (n = 7), and Driyorejo (7°21’11.1”S 112°37’43.9”E) (n = 9) (Figure-1). Identification of *aer-A* gene and antimicrobial resistance of *A. hydrophila* were carried out at the Gamma Scientific BioLab, Malang, and Institute of Tropical Diseases.

### Isolation and identification

A total of 153 freshly dead milkfish were collected from 16 locations in Gresik based on rearing ponds. All samples were transferred to the laboratory in a polyethylene bag and icebox. Swab gills were pre-enriched in alkaline peptone water (Oxoid CM1028, UK) at 37°C for 24 h, then cultured on blood agar (BA) (Oxoid CM55) at 28°C for 24 h. Growth colonies on BA media with hemolytic characteristics were then purified on trypticase soy agar (Oxoid CM131) at 37°C for 18–24 h. Colonies were also subcultured on Rimler-Shotts (RS) (Thermo Fisher Scientific Pte. Ltd., Australia) media at 37°C for 24 h, and then the yellow-colored colonies were further identified according to biochemical characteristics using potassium cyanide (KCN), catalase, oxidase, lipase, gelatinase, and protease tests [10].

### Aerolysin gene identification

The *aer-A* gene was identified using the primers forward 5’-CAAGAACAAGTTCAAGTGGCCA-3’ and reverse 5’–ACGAAGGTGTGGTTCCAGT-3’ (GoTaq^®^ Green DNA polymerase, Promega Corp, USA). A total of 12.5 μL of polymerase chain reaction (PCR) buffer mixture was prepared that consisted of 2.5 μL of magnesium chloride (25 mM), 0.5 μL of deoxynucleoside triphosphate mixture (200 μM), 2.5 μL of the forward primer (12 μM), 2.5 μL of the reverse primer (12 μM), 12.5 mM (GoTaq^®^ Green DNA polymerase, Promega Corp), and 3 μL of DNA template [11].

Furthermore, the DNA amplification process for the *aer-A* gene was initiated by denaturation at 95°C for 5 min, annealing at 59°C for 30 s, elongation at 72°C for 30 s, and final elongation at 72°C for 7 min. All stages were repeated for 50 cycles. The amplification results were then visualized with agarose gel electrophoresis of 0.8% buffer, tris-acetate-ethylenediaminetetraacetic acid, and 100 bp marker (GoTaq^®^ Green DNA polymerase, Promega Corp). The *aer-A* gene-positive samples were revealed at an amplicon length of 309 bp [11].

### Antimicrobial resistance evaluation

*A. hydrophila* isolates were inoculated in tryptic soy broth, then incubated at 35°C for 24 h. Broth suspension was inoculated in the Mueller-Hinton broth (Oxoid CM0405), then the turbidity level was adjusted to the 0.5 McFarland standard. Thereafter, the isolates were streaked on the Mueller-Hinton agar (Oxoid CM0337) followed by disk (Oxoid) placement and incubated at 37°C for 24 h. The antibiotic disks used were amoxicillin (AML, 25 μg), cefotaxime (CTX, 30 μg), chloramphenicol (C, 30 μg), ciprofloxacin (CIP, 5 μg), erythromycin (E, 15 μg), gentamycin (CN, 10 μg), imipenem (IPM, 10 μg), kanamycin (K, 30 μg), nalidixic acid (NA, 30 μg), streptomycin (S, 10 μg), tetracycline (TE, 30 μg), and trimethoprim (SXT, 25 μg). The evaluation was performed under the Clinical and Laboratory Standards Institute guidelines for the following antibiotics: AML, CTX, C, E, CN, IPM, S, and TE [12]. Meanwhile, CIP was evaluated as per the guideline of the European Committee on Antimicrobial Susceptibility Testing [13], K was evaluated as per the guideline of the Comite de lʼAntibiogramme de la Societe Francaise de Microbiologie [14], NA and SXT were evaluated as per the guideline of the French Society of Microbiology [15].

### Statistical analysis

The data were descriptively evaluated and presented in tables.

## Results

Of the 153 collected milkfish samples, 35 (22.9%) were confirmed positive for *A. hydrophila* infection based on the biochemical evaluation of KCN and oxidase reactions. The isolates were followed by a PCR test to reveal the *aer-A* gene with 309 bp amplicon size and 100 bp ladder. A total of 22 (62.9%) of the 35 samples were confirmed positive for the *aer-A* gene (Table-1).

**Table 1:** Prevalence of *A. hydrophila* in the examined samples.

Distributions	Suspected isolates (%)	BA	TSA	RS	KCN	Catalase	Oxidase	Lipase	Gelatinase	Protease	Confirmed *Aeromonas hydrophila* (%)	*aer-A* gene positive
Dukun (n = 7)	4 (2.6)	4	4	3	3	3	3	0	0	2	3 (2)	2 (5.7)
Panceng (n = 8)	4 (2.6)	4	4	4	4	3	4	0	2	4	4 (2.6)	2 (5.7)
Ujung Pangkah (n = 11)	8 (5.2)	7	6	5	5	5	5	2	2	5	5 (3.3)	3 (8.6)
Sidayu (n = 15)	7 (4.6)	5	5	5	5	5	5	3	2	4	5 (3.3)	2 (5.7)
Bungah (n = 14)	7 (4.6)	6	4	4	4	4	4	0	1	4	4 (2.6)	2 (5.7)
Manyar (n = 11)	3 (2)	3	3	3	3	3	3	1	1	3	3 (2)	2 (5.7)
Gresik (n = 12)	4 (2.6)	2	2	2	2	2	2	1	1	1	2 (1.3)	2 (5.7)
Kebomas (n = 7)	0 (0)	0	0	0	0	0	0	0	0	0	0 (0)	0 (0)
Duduk Sampeyan (n = 8)	3 (2)	3	3	2	2	2	2	0	1	1	2 (1.3)	1 (2.9)
Cerme (n = 11)	3 (2)	3	2	2	2	2	2	2	2	2	2 (1.3)	2 (5.7)
Benjeng (n = 10)	0 (0)	0	0	0	0	0	0	0	0	0	0 (0)	0 (0)
Balong Panggang (n = 7)	1 (0.7)	1	0	0	0	0	0	0	0	0	0 (0)	0 (0)
Wringinanom (n = 8)	2 (1.3)	2	1	1	1	1	1	0	1	1	1 (0.7)	1 (2.9)
Menganti (n = 8)	1 (0.7)	1	1	1	1	1	1	0	0	1	1 (0.7)	0 (0)
Kedamean (n = 7)	4 (2.6)	4	2	1	1	1	1	0	1	1	1 (0.7)	1 (2.9)
Driyorejo (n = 9)	2 (1.3)	2	2	2	2	2	2	1	1	1	2 (1.3)	2 (5.7)
Total (n = 153)	53 (34.6)	47 (30.7)	39 (25.5)	35 (22.9)	35 (22.9)	34 (22.2)	35 (22.9)	10 (6.5)	15 (9.8)	30 (19.6)	35 (22.9)	22 (62.9)

BA=Blood agar, TSA=Trypticase soy agar, RS=Rimler-Shotts, KCN=Potassium cyanide. Aerolysin (*aer*-A) gene investigation at 309 bp in confirmed samples

The antibiotic susceptibility investigation reported that all samples were susceptible to CIP and IPM. Meanwhile, the evidence of the highest antibiotic resistance was reported in AML (62.9%), TE (60%), S (54.3%), and CTX (51.4%). The lowest resistance was reported to CN (31.4%), K (28.6%), E (25.7%), C (20%), and SXT (14.3%) (Table-2).

**Table 2:** Antimicrobial resistance profile of *Aeromonas hydrophila* isolates (n = 35).

Antimicrobials	Resistant (%)	Intermediate (%)	Sensitive (%)
AML 25	22 (62.9)	2 (5.7)	11 (31.4)
CTX 30	18 (51.4)	n/a	17 (48.6)
C 30	7 (20)	n/a	28 (80)
CIP 5	n/a	n/a	35 (100)
E 15	9 (25.7)	4 (11.4)	22 (62.9)
CN 10	11 (31.4)	3 (8.6)	21 (60)
IPM 10	n/a	n/a	35 (100)
K 30	10 (28.6)	4 (11.4)	21 (60)
NA 30	n/a	5 (14.3)	30 (85.7)
S 10	19 (54.3)	5 (14.3)	11 (31.4)
TE 30	21 (60)	n/a	14 (40)
SXT 25	5 (14.3)	n/a	30 (85.7)

AML=Amoxicillin, CTX=Cefotaxime, C=Chloramphenicol, CIP=Ciprofloxacin, E=Erythromycin, CN=Gentamycin, IPM=Imipenem, K=Kanamycin, NA=Nalidixic acid, S=Streptomycin, TE=Tetracycline, SXT=Trimethoprim

## Discussion

This study evaluated 153 samples and reported a prevalence rate of 22.9%. This prevalence remained in a low category compared to the prevalence rate of 80% in tilapia in Egypt [16]. Another recent study in Egypt reported a lower percentage of *A. hydrophila* from Nile tilapia (41%) [17], 46.4% in freshwater aquaculture in Vietnam [18], 75.4% from seafood in South Korea [19], 53.3% in fresh Mugil flesh [20], 90.16% in Sukabumi, 90.05% in Surabaya, and 88.31% in Jepara isolate [21]. *A. hydrophila* infection is one of the main focuses and has a crucial impact on the aquaculture sector. This bacterium is pathogenic and causes Motile *Aeromonas* Septicemia (MAS) disease in freshwater fish culture in tropical areas [22]. In general, the clinical symptoms in the organs as a hemorrhagic septicemia manifestation include ulceration in the eyes, gills, fins, scales, and muscles in the abdominal area. Moreover, hemorrhage is often found in the liver, spleen, intestines, and kidneys when necropsy is performed [23].

We revealed the characteristics of cream-colored bacteria in colonies, rod-shaped cell morphology, Gram-negative, producing catalase enzymes, oxidases, fermenting lactose, and several proteases based on the biochemical *A. hydrophila* identification (Table-1). A previous study reported the same findings that *A. hydrophila* is also motile, catalyzes D-mannitol, forms a hemolysis zone on BA media, and properly grows on RS and KCN media [24].

*A. hydrophila* contains exoenzymes that are encoded by lipase, nuclease, and serine protease genes and contains exotoxin derivatives called aerolysin. Our study identified *aer-A* genes in 22 of 35 isolates (62.9%) (Table-1). The previous studies indicated *aer-A* gene in 96% of isolates from catfish [25], 37.5% of clinical isolates in Canada [26], 62.7% of isolates from zebrafish [27], 28.8% of clinical isolates in Tokyo and Kanagawa Prefecture, Japan [28], and 33.3% of isolates from Nile tilapia in Egypt [17]. Aerolysin is known to be highly virulent and increases the pathogenicity of *A. hydrophila*. An *in vitro* study revealed that epithelial integrity disorders may occur due to aerolysin lyses tight junction protein as a gastrointestinal mucosal barrier [29]. However, studies on the molecular mechanisms of aerolysin-induced cell lysis remained limited. Some evidence of aerolysin-related pathogenesis is limited to reporting the possibility of *A. hydrophila* crossing the gastrointestinal barrier and systemically infecting other organs [30].

Appropriate drug selection as a curative method for fish diseases and the aquaculture sector must consider the risk of antimicrobial residues to human health. Antimicrobial resistance severity needs to be evaluated periodically to assess possible susceptibility [31]. The emergence of resistant bacteria in fish is often found due to the widespread use of antimicrobials and unregulated according to infectious disease control protocols. Resistance genes can increase through horizontal gene transmission and increase pathogenicity in humans [32]. This study revealed that only CIP and IPM were susceptible to all isolates. Meanwhile, we indicated the presence of isolates resistant to AML, TE, S, CTX, CN, K, E, C, and SXT (Table-2). We conducted the first study on milkfish in Gresik to evaluate the possible antimicrobial resistance of *A. hydrophila* isolates. The previous studies reported that *A. hydrophila* was sensitive to CIP, azithromycin, and CN [33], ampicillin and cephalexin [34], and AML, ampicillin, lincomycin, novobiocin, oxacillin, penicillin, and SXT in combination with sulfamethoxazole and rifampicin [35].

Various antimicrobials treat disease by selectively inhibiting protein synthesis activity on bacterial ribosomes. As is known, the function of the ribosome is to translate genetic information encoded by mRNA and produce protein molecules that have been specified by mRNA [36]. The protein that is produced is responsible for all the cellular activities of bacteria. Each antimicrobial inhibits protein synthesis by a different mechanism, depending on the antimicrobial receptor on the bacterial ribosome [37]. Streptomycin selectively binds to the 30S subunit and this binding blocks protein synthesis at the initiation stage [38]. The C receptor on the ribosome is on the 50S subunit, where its binding inhibits peptide bond formation [39]. TE has receptors on the 30S subunit and then blocks the binding of aminoacyl-tRNA to the ribosomal mRNA complex at the A-site [40].

Antimicrobial resistance is caused by various factors, but the most crucial is the resistance gene prevalence and antibiotic use extension. Antibiotic misuse, such as therapeutic errors, incomplete or prolonged use, and inadequate doses, often causes resistance [41]. Bacteria have resistance traits that can be natural because these traits have been inherited by previous bacterial species or acquired resistance, which is a manifestation of resistance gene mutations that are present among bacteria [42]. Antibiotic action that normally inactivates the target bacterial protein becomes ineffective due to mutations that remove the protein or mutations that increase enzyme production in the target antibiotic. Resistant genes can also arise through gene exchange between bacteria through conjugation involving plasmids, transduction involving bacteriophages to transport resistance genes, and transposition involving transposons or transformations that produce new genotypes through transposons or transformations that produce new genotypes through genetic recombination. Resistant genes, through these various mechanisms, can move from one bacterium to another and lead to the rapid spread of resistance [43, 44].

## Conclusion

The prevalence of *A. hydrophila* from milkfish in Gresik, Indonesia, is 22.9% and 62.9% of positive isolates confirmed the *aer-A* gene. Meanwhile, antimicrobial resistance was reported to be expressed in AML, TE, S, CTX, CN, K, E, C, and SXT. In addition, only CIP and IPM were susceptible to all isolates. However, identifying the pathological lesions in the liver, spleen, and intestine of milkfish in MAS need to be further disclosed. Therefore, the collaboration of veterinarians and aquaculture managers is necessary to mitigate the risk of spreading the disease, particularly, the importance of epidemiological studies to control diseases, curative actions taken, and their impact on public health.

## Authors’ Contributions

MTEP, FF, and DKW: Conceptualized and designed the study. MTEP, FF, DKW, and AP: Collected the samples. FF and DKW: Performed sample identification. SK: Data analysis. AP and SK: Visualization and validation of tables and figures. FF, SK, and SC: Drafted the manuscript. MTEP and SC: Revised and submitted the manuscript. All authors have read and approved the final manuscript.
